# Prenatal Stress, Fearfulness, and the Epigenome: Exploratory Analysis of Sex Differences in DNA Methylation of the Glucocorticoid Receptor Gene

**DOI:** 10.3389/fnbeh.2016.00147

**Published:** 2016-07-12

**Authors:** Brendan D. Ostlund, Elisabeth Conradt, Sheila E. Crowell, Audrey R. Tyrka, Carmen J. Marsit, Barry M. Lester

**Affiliations:** ^1^Department of Psychology, University of UtahSalt Lake City, UT, USA; ^2^Department of Psychiatry, University of Utah School of MedicineSalt Lake City, UT, USA; ^3^Mood Disorder Research Program and Laboratory for Clinical and Translational Neuroscience, Butler HospitalProvidence, RI, USA; ^4^Department of Psychiatry and Human Behavior, Warren Alpert Medical School of Brown UniversityProvidence, RI, USA; ^5^Department of Environmental Health, Rollins School of Public Health, Emory UniversityAtlanta, GA, USA; ^6^The Brown Center for the Study of Children at Risk, Brown UniversityProvidence, RI, USA; ^7^Department of Pediatrics, Warren Alpert Medical School of Brown UniversityProvidence, RI, USA

**Keywords:** DNA methylation, fearfulness, prenatal stress, sex differences, glucocorticoid receptor gene, temperament

## Abstract

Exposure to stress *in utero* is a risk factor for the development of problem behavior in the offspring, though precise pathways are unknown. We examined whether DNA methylation of the glucocorticoid receptor gene, *NR3C1*, was associated with experiences of stress by an expectant mother and fearfulness in her infant. Mothers reported on prenatal stress and infant temperament when infants were 5 months old (*n* = 68). Buccal cells for methylation analysis were collected from each infant. Prenatal stress was not related to infant fearfulness or *NR3C1* methylation in the sample as a whole. Exploratory sex-specific analysis revealed a trend-level association between prenatal stress and increased methylation of *NR3C1* exon 1F for female, but not male, infants. In addition, increased methylation was significantly associated with greater fearfulness for females. Results suggest an experience-dependent pathway to fearfulness for female infants via epigenetic modification of the glucocorticoid receptor gene. Future studies should examine prenatal stress in a comprehensive fashion while considering sex differences in epigenetic processes underlying infant temperament.

## Introduction

The antecedents of internalizing symptoms in adulthood may be evident in the first months of life. Exaggerated fearfulness in infancy is one such precursor to the internalizing spectrum (Buss and McDoniel, 2016). Fearfulness in infancy manifests as a profile of negative reactivity characterized by excessive fussiness and motor agitation in response to novelty (Kagan and Snidman, 1991). As toddlers, these children are prone to experiencing distress and timidity toward unfamiliar people, objects, or situations (Kagan, 2013). This dispositional expression of negative affect remains moderately stable through childhood and into young adulthood, and is implicated in the development of internalizing psychopathology, particularly social anxiety (Caspi et al., 1996; Biederman et al., 2001; Hirshfeld-Becker et al., 2007; Chronis-Tuscano et al., 2009; Buss, 2011). However, the origins of fearfulness in infancy remain unclear, though heritable and prenatal mechanisms are likely contributors. Stressful events experienced by an expectant mother during pregnancy and subsequent alterations in the infant’s stress physiology may account for some of the observed differences in fearful temperament.

Stressful life events can take many forms and may include experiences of adversity or psychopathology. Mothers who are depressed are more likely to report stressful life events (e.g., relationship conflict, unemployment), compared to their non-depressed counterparts (Ertel et al., 2011). The effects of stress exposure on an expectant mother can have an early and enduring impact on her child’s functioning (Sandman et al., 2011). Exposure to prenatal stress has been associated with increased fearful behavior (Bergman et al., 2007, 2008; Davis et al., 2007; Werner et al., 2007) and stress reactivity (Yehuda et al., 2005; Oberlander et al., 2008; Davis et al., 2011), placing these children at risk for developing internalizing symptoms later in life (Crowell and Kaufman, in press). For instance, longitudinal work from Davis and Sandman (2012) showed that children exposed to maternal anxiety and stress *in utero* exhibit more internalizing symptoms across childhood, with rank order stability persisting into adolescence (O’Donnell et al., 2014).

Research over the past decade has sought to explicate the mechanisms underlying the association between stressful events experienced during pregnancy and individual differences in fearful temperament in infancy. Cortisol, a glucocorticoid product of hypothalamic-pituitary-adrenal (HPA) axis activity, has been examined as a possible mediator of the association between prenatal stress exposure and child functioning with promising, yet inconclusive, results (for review see Glover et al., 2010). Elevated basal cortisol and cortisol reactivity are associated with fearfulness and inhibition in childhood (Kagan et al., 1987; Nachmias et al., 1996; Schmidt et al., 1997; Spangler and Schieche, 1998; Buss et al., 2004; Pérez-Edgar et al., 2008). Similar effects have been reported among adults, with inhibited individuals demonstrating greater cortisol reactivity during a stress test (Tyrka et al., 2008). These effects have also been found in animal models. For instance, rat pups exposed to maternal prenatal stress display fearfulness and exaggerated emotionality as a consequence of glucocorticoid-facilitated up-regulation of corticotropin-releasing hormone in the amygdala (for discussion see Maccari et al., 2014).

Epigenetic modifications of HPA-related genes are a likely candidate to account for some of the individual differences in fearfulness through stable changes to the functioning of the individual’s stress response system (Turecki and Meaney, 2016). Epigenetic modifications alter gene expression without changing the nucleotide sequence of DNA. One form of epigenetic modification, DNA methylation, involves the addition of a methyl group to a cytosine in the context of a cytosine and guanine dinucleotide (for reviews see Champagne, 2010; Meaney, 2010), and is responsible, in part, for gene expression control. DNA methylation of the glucocorticoid receptor gene, *NR3C1*, has received attention due to its role in HPA axis functioning. Sustained HPA axis activity is, in part, modulated by glucocorticoid receptors; as cortisol binds with glucocorticoid receptors, the amount of cortisol is reduced and subsequent secretion diminishes. Increased methylation of *NR3C1* reduces glucocorticoid receptor gene expression via attenuated transcription activity, thereby limiting the availability of glucocorticoid receptors. As a result, cortisol has fewer glucocorticoid receptors with which to bind and thus remains at higher levels, with implications for long-term basal cortisol levels and cortisol reactivity.

Oberlander et al. (2008) found that variations in prenatal maternal mood were related to increased methylation of exon 1F of the promoter region of *NR3C1* in the infant, which in turn was associated with increased cortisol response at 3 months. In support of these findings, research from Conradt et al. (2013) showed that infants whose mothers reported elevated depression in pregnancy and had greater placental methylation of exon 1F of the promoter region of *NR3C1* demonstrated impairments in neurobehavioral functioning (i.e., self-regulation, lethargy, tonicity). Methylation of *NR3C1* at exons 1D and 1F has also been associated with increased internalizing behaviors among preschoolers exposed to early adversity, suggesting the relevance of neuroendocrine-mediated epigenetic processes for the development of psychopathology in early life (Parade et al., 2016). Taken together, the extant research suggests that experiences of prenatal stress by an expectant mother may be associated with emerging behaviors such as fearfulness (e.g., inhibition, hypervigilance, distress) in the infant via epigenetic processes that alter activity of the HPA axis.

Sex-specific differences are often under-investigated in research on temperament, particularly in infancy. With respect to fear, females tend to display more fearfulness in infancy compared to their male peers (Gartstein and Rothbart, 2003; Else-Quest et al., 2006). Females tend to report more neuroticism/negative emotionality compared to males from late childhood through adulthood (Costa et al., 2001), and are at increased risk for internalizing psychopathology (Kessler et al., 2005). Recent findings in behavioral epigenetics highlight how epigenetic pathways may differ between males and females. Lesseur et al. (2014) found that among male, but not female, newborns, DNA methylation of the leptin promoter was associated with classification in a neurobehavioral profile characterized by lethargy and hypotonicity. In addition, prenatal maternal depression has been shown to predict increased methylation of *NR3C1* in 2-month-old male infants, but not their female counterparts (Braithwaite et al., 2015). Examining potential sex-specific effects may elucidate important individual differences in the origins of temperament, thereby providing a more nuanced perspective on the development of risk for internalizing problems.

### Present Study

The aims of the present study were to: (1) examine whether a woman’s experience of stressful events during her pregnancy was related to individual differences in infant fearful temperament at 5 months; (2) test whether DNA methylation of the promoter region of the glucocorticoid receptor gene *NR3C1* exon 1F was associated with stressful events during pregnancy and fearful temperament in infancy; and (3) test DNA methylation of the promoter region of the glucocorticoid receptor gene *NR3C1* exon 1F as a mediator explaining any associations between stressful life events during pregnancy and fearful temperament in infancy. Guided by previous research, we hypothesized that a woman’s experience of stress during pregnancy would be related to DNA methylation of her infant’s glucocorticoid receptor gene, which in turn would be associated with fearful temperament. Moreover, we examined whether these findings differed based on the sex of the infant. Examination of sex-specific differences was considered exploratory.

## Materials and Methods

### Participants

Mothers and their 5-month-old infants were part of the Rhode Island Child Health Study, an existing cohort recruited through a local hospital in Providence, Rhode Island. Mothers from the original study were recruited in accordance with the Institutional Review Boards of Dartmouth College and Women and Infants Hospital. All mothers gave written informed consent prior to participation.

Only singleton, full-term (≥37 weeks gestational age) infants were included in the study. Other exclusion criteria included maternal age <18 years or a life-threatening medical complication of the mother, and congenital or chromosomal abnormality of the infant. Mother-infant dyads were invited into the laboratory when infants were 5 months old. The current sample consisted of mother-infant dyads for whom we had complete data on all variables of interest. Infants who received a diagnosis of intrauterine growth restriction were excluded from analysis. Mothers in the current sample identified as European American (69%), African American (13%), Hispanic (6%), Asian (2%), or “other” (9%), and had a mean age of 30.4 years (range = 20–40 years). The sample included 87 infants (45 females) with an average age of 19.8 weeks (range = 15–26 weeks) at the time of assessment. Sample characteristics are shown in Table 1.

**Table 1:** **Demographic characteristics of current sample**.

	Mean/Percentage	SD	Range
**Age**
Mother (years)	30.39	4.75	20–40
Gestational age	39.28	0.75	37–41
Infant (weeks)	19.84	2.63	15–26
**Household income**
$0–24,999	14%
$25,000–49,999	18%
$50,000–79,999	15%
>80,000	48%
Did not report	5%
**Maternal Education**
High school graduate or less	14%
Some college/Junior college	26%
College graduate or beyond	60%
Married	70%
**Ethnicity**
European American	69%
African American	13%
Hispanic	6%
Asian	2%
Other	9%

### Measures

#### Stressful Experiences in Pregnancy

Experiences of stress during pregnancy were assessed at the 5-month laboratory visit using the Significant Life Events (SLE) questionnaire (see Appendix in Supplementary Material). The SLE questionnaire is a 16-item self-report measure developed in our laboratory that retrospectively assesses stressful life events that may occur during pregnancy (e.g., emotional abuse, death of a partner, medical complications). This questionnaire also assesses when an event occurred, the frequency of occurrence, and the woman’s subjective experience of the event. These subcomponents, however, were not addressed in the current analysis due to lack of variability. Approximately 37% of the sample (*n* = 32) experienced at least one SLE during their pregnancy, and *n* = 9 experienced two or more; we dichotomized to compare participants with any SLE to those with none.

#### Fearful Temperament at 5 Months

Temperamental differences in fear in early infancy were examined using the Infant Behavior Questionnaire—Revised (IBQ-R; Gartstein and Rothbart, 2003). The IBQ-R is a 191-item maternal report measure that assesses 14 dimensions of temperament in 3–12 month-old infants. This study focused exclusively on the Fear scale of the IBQ-R. This scale assesses temperamental differences in inhibition toward novel objects and/or individuals, as well as distress to sudden changes in stimulation. The IBQ-R demonstrated satisfactory internal consistency within the current sample (*α* = 0.83). The Fear scale has also demonstrated strong inter-rater reliability between primary and secondary caregivers (*r* = 0.75; Gartstein and Rothbart, 2003).

### DNA Methylation of Glucocorticoid Receptor Gene (*NR3C1*) at 5 Months

#### Buccal Sample Collection, DNA Isolation, and Bisulfite Modification

Buccal-derived DNA was collected from saliva samples using the Oragene-DNA saliva collection system. Buccal cells were taken from the infants’ cheeks using a small swab. The swabs were then placed in a collection tube and sealed, releasing a stabilizing solution into the collected sample to allow for processing of the sample at a later period. Batches of sample collection tubes were sent to (blinded for review) College for DNA isolation. DNA was isolated from the collection tubes following the Oragene methods. Purified DNA was quantified using a ND-1000 spectrophotometer (Nanodrop, Wilmington, DE, USA), and DNA samples (500 ng) were bisulfite-modified using the EZ DNA Methylation Kit (Zymo Research, CA, USA) and stored at −20°C.

#### Bisulfite Pyrosequencing DNA Methylation Analysis

Pyrosequencing, which allows for quantitative assessment of DNA methylation in short sequence regions, was performed on PCR product amplified from bisulfite modified DNA as described previously (blinded for review). The primers for amplification were Forward: 5′-TTT TTT TTT TGA AGT TTT TTT A-3′ and Reverse: 5′-Biotin-CCC CCA ACT CCC CAA AAA-3′. The first sequencing primer was designed to sequence the first five CpG sites (5′-GAG TGG GTT TGG AGT-3′), and the second sequencing primer was designed to sequence the following eight CpG sites (5′- AGA AAA GAA TTG GAG AAA TT-3′) for a total of 13 sites sequenced.

The percent methylation at each of the 13 CpG sites of *NR3C1* was quantified using the Pyro Q-CpG software, version 1.0.11 (Qiagen). Bisulfite conversion controls were included on each sequencing read. In order for the sample’s methylation extent to be called, the bisulfite conversion rate must be >93%, and for all samples examined the conversion rate was >95%. All assays were performed in triplicate on the same bisulfite converted DNA template on all samples, and if any of the repeats differed by >10% those assays on that sample were repeated. To prevent batch effects from bisulfite treatments interfering with the analysis, samples were randomized across batches. Percent methylation was averaged across the 13 CpG sites of *NR3C1*.

### Analytical Approach

Factorial analyses of variance (ANOVA) was used to examine whether a stressful life event during pregnancy, infant sex, and their interaction were associated with infant fearful temperament at 5 months. The same approach was initially used to examine whether the experience of a stressful event during pregnancy, infant sex, and their interaction were associated with DNA methylation of *NR3C1*. The variances, however, significantly differed between the groups (*F*_(3,65)_ = 3.23, *p* = 0.03), thus violating the assumption of homogeneity of variance. Instead, an independent samples *t*-test was run to determine if an infant’s DNA methylation of *NR3C1* differed based on prenatal exposure to a stressful event. This approach was first conducted using the entire sample, and then in exploratory analyses based on infant sex. Results of the independent samples *t*-tests reported below do not assume equal variance. The association between DNA methylation of *NR3C1* and infant fearfulness at 5-months was examined using Spearman rank correlations, due to the positive skewness of both variables.

## Results

### Preliminary Analyses

Descriptive statistics for variables of interest are presented in Table 2. Data were examined for outliers and violations of normality prior to primary analyses. As reported above, the SLE in pregnancy variable was dichotomized to represent whether or not a mother experienced a significant life event during her pregnancy. Covariates related to SLE during pregnancy, DNA methylation, and fearful temperament in infancy were examined prior to the primary analyses. Covariates included maternal age, maternal ethnicity, gestational age, birth weight, and infant age in weeks at the assessment. None of the covariates were associated with the variables of interest for females (all *p*’s > 0.10) or males (all *p*’s > 0.16). Maternal sensitivity at 5 months, assessed during the initial play episode of the Still-Face Paradigm (Tronick et al., 1978), was also examined as a covariate. Two factors, responsiveness/appropriate touch and acceptance/non-demandingness, emerged from the principal components analysis; together, these factors accounted for approximately 80% of the variance (see Conradt et al., 2016 for description of sensitivity coding and factor analysis of maternal behaviors). Mothers who experienced a stressful life event during pregnancy did not differ in regards to responsiveness/appropriate touch (*F*_(1,78)_ = 1.74, *p* = 0.19) or acceptance/non-demandingness (*F*_(1,78)_ = 0.08, *p* = 0.78), compared to their counterparts who did not experience a stressful event. DNA methylation of *NR3C1* at exon 1F was not significantly associated with responsiveness/appropriate touch (*ρ* = −0.12, *p* = 0.32) or acceptance/non-demandingness (*ρ* = −0.07, *p* = 0.57). Infant fearfulness was not significantly associated with responsiveness/appropriate touch (*ρ* = −0.05, *p* = 0.68); the association between infant fearfulness and acceptance/non-demandingness was trending toward significance (*ρ* = −0.20, *p* = 0.07). There was no significant difference in methylation of *NR3C1* between male (*M* = 2.16, *SD* = 0.64) and female (*M* = 2.10, *SD* = 0.79) infants, *t*_(68)_ = 0.38, *p* = 0.71. There was also no difference in fearful temperament between male (*M* = 2.41, *SD* = 1.25) and female (*M* = 2.66, *SD* = 0.90) infants, *t*_(86)_ = −1.11, *p* = 0.27.

**Table 2:** **Descriptive statistics for variables of interest**.

Variables	*n*	*M*	SD	Range
SLE in pregnancy^a^
- None	54
- 1st trimester^b^	19
- 2nd trimester	4			
- 3rd trimester^c^	9			
DNA methylation	70	2.13	0.71	1.26–4.41
Fear (IBQ-R)	87	2.53	1.09	1.25–6.87
Infant sex
- Males	42
- Females	45

*^a^n = 32 (37% of the sample) women reported experiencing a SLE during pregnancy, with n = 9 women reporting multiple SLE. ^b^n = 4 women experienced >1 SLE during the 1st trimester. ^c^n = 1 women experienced >1 SLE during the 3rd trimester*.

### Primary Analyses

#### Is a Women’s Experience of Stress in Pregnancy Associated with her Infant’s Fearful Temperament?

**Figure 1:**
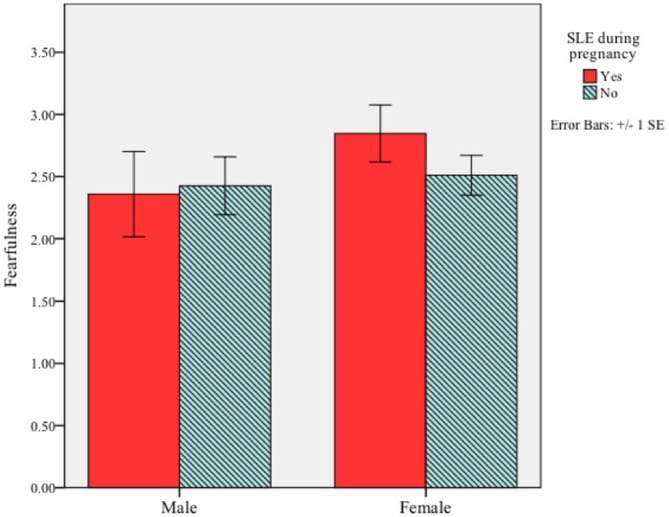
**Interaction between infant sex and maternal significant life events (SLE) during pregnancy on fearful temperament at 5 months of age**. The interaction did not reach significance (*p* = 0.42).

First, we examined whether a mother’s experience of a significant life event during her pregnancy was associated with fearfulness in her infant. There was no main effect for prenatal exposure to a stressful life event (*F*_(1,86)_ = 0.35, *p* = 0.59) or infant sex (*F*_(1,86)_ = 1.59, *p* = 0.25) on infant fearfulness. Moreover, the interaction between these variables was not significant, *F*_(1,86)_ = 0.79, *p* = 0.42 (Figure 1).

#### Is a Woman’s Experience of Stress in Pregnancy Related to DNA Methylation in her Infant?

**Figure 2:**
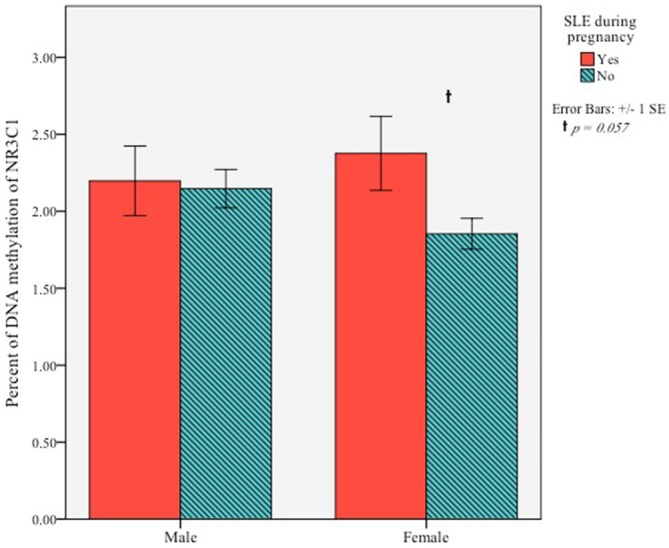
**Percent of DNA methylation of *NR3C1* exon 1F for 5-month-old infants based on whether their mother experienced of a SLE during pregnancy**.

Next, we examined the association between maternal SLE during pregnancy and methylation of *NR3C1* at exon 1F. An independent samples *t*-test was conducted to determine if infants’ DNA methylation at 5 months differed based on prenatal exposure to maternal stress. There was no sample-wide effect, *t*_(37.77)_ = −1.46, *p* = 0.15. Exploratory analyses testing for within sex effects showed that among females, DNA methylation was greater for infants whose mother experienced a SLE in pregnancy (*M* = 2.38, *SD* = 0.99) compared to those whose mothers did not (*M* = 1.85, *SD* = 0.41), *t*_(21.41)_ = −2.01, *p* = 0.057, although this effect was trending toward significance. There was no effect for males, *t*_(33)_ = −0.20, *p* = 0.84 (Figure 2).

#### Is DNA Methylation Associated with a Fearful Temperament in Infancy?

**Figure 3:**
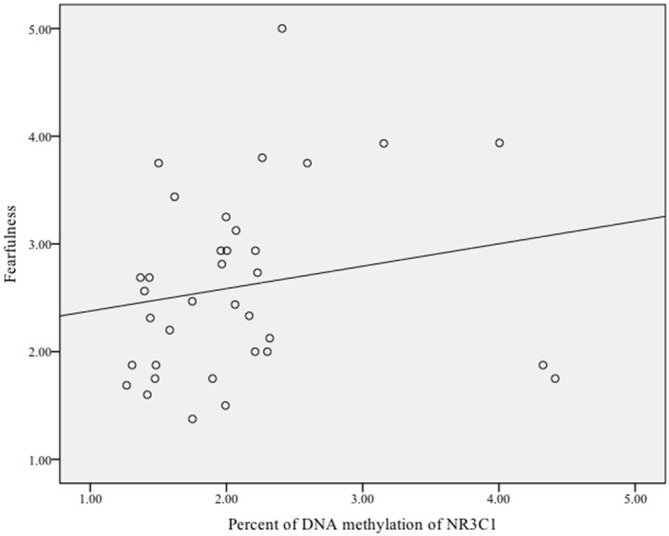
**Scatterplot of the association between DNA methylation of the glucocorticoid receptor gene (*NR3C1*) and fearful temperament at 5 months among female infants**.

**Figure 4:**
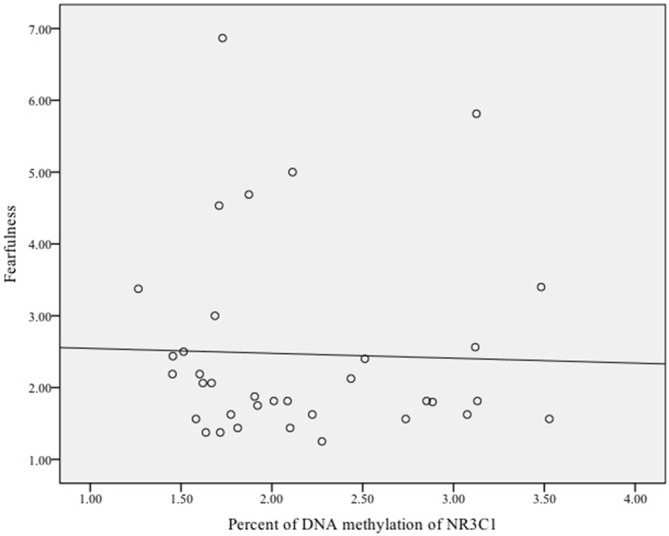
**Scatterplot of the association between DNA methylation of the glucocorticoid receptor gene (*NR3C1*) and fearful temperament at 5 months among male infants**.

Finally, we examined whether DNA methylation of *NRC31* was related to infant fearfulness. There was no sample-wide association between methylation of* NR3C1* and fearful temperament, *ρ* = 0.03, *p* = 0.78. Exploratory analyses among females showed that more methylation of the glucocorticoid receptor gene was related to greater fearfulness in infancy, *ρ* = 0.35, *p* = 0.04 (Figure 3). Once again, there was no effect for males, *ρ* = −0.11, *p* = 0.51 (Figure 4).

## Discussion

The goal of the present study was to identify epigenetic processes associated with the developmental origins of fearful temperament. We examined epigenetic modifications of the glucocorticoid receptor gene, *NR3C1*, as a mechanism linking experiences of prenatal stress by an expectant mother to fearfulness in her infant. Contrary to expectations, we did not find any associations between maternal prenatal stress, DNA methylation, and fearful temperament in the overall sample. However, exploratory analyses did find that in female infants, experiences of stress during pregnancy were related to increased methylation of *NR3C1* at exon 1F, although this was a trend-level effect. Methylation of *NR3C1* was also significantly associated with greater fearfulness in infancy among females.

An expectant mothers’ experience of stress was not related to her infant’s fearful temperament. This was unexpected given the replicability of this finding across research groups (Bergman et al., 2007; Davis et al., 2007; Werner et al., 2007). Prior work has employed a variety of behavioral (e.g., Laboratory Temperament Assessment Battery) and maternal report (e.g., fear subscale of the IBQ-R) techniques to assess fearful behavior, and all converged on the same result: increased prenatal stress is associated with increased fearfulness among low-risk infants. Thus, it is unlikely that the absence of an effect in the present study is due to the temperament instrument used, sample size, or unique characteristics of this sample. It may also be that the type and/or timing (e.g., trimester of pregnancy when experienced, duration of stress) of a stressful event is relevant to the development of fearful behavior. For instance, Bergman et al. (2007) found that events pertaining to relationship strain (e.g., “your partner was emotionally cruel to you”, “you had a serious argument with your partner”) were associated with fearful behavior in infancy (p. 1459, Bergman et al., 2007), whereas Davis et al. (2007) and Werner et al. (2007) found that variations in prenatal maternal mood (i.e., anxiety, depression) were related to increased fearfulness. It is possible that the inclusion of more stressors associated with relationship strain or prenatal maternal mood would have yielded a stronger association between prenatal stress and fearfulness in infancy. Moreover, examining these associations within a sample of higher-stressed women, such as women experiencing emotion dysregulation, may have yielded a stronger association between maternal prenatal stress and infant fearfulness.

Exposure to stress prenatally has been shown to have an early and enduring impact on a child’s behavioral and emotional development. We found a trend of increased methylation of *NR3C1* at exon 1F in 5-month-old female infants whose mothers experienced a stressful event during pregnancy. Given the importance of sex differences in the child development literature and the dearth of research on sex effects in the behavioral epigenetics literature, we suggest that more attention be paid to sex differences in studies of epigenetic phenomenon in child behavioral development. Moreover, our findings compliment prior work suggesting that methylation of *NR3C1* at exon 1F may differ depending on the type of prenatal stress experienced by male (e.g., maternal depression; Braithwaite et al., 2015) or female infants. The sex-specific effects identified in the present study may point to one molecular pathway involved in the development of sex differences in internalizing behavior. Although no differences were observed in the current sample, prior work has demonstrated a small sex difference in infant fearful temperament (Gartstein and Rothbart, 2003). Across the lifespan, females are at higher risk than males for developing anxiety and mood disorders (Kessler et al., 2005).

Findings from the present study may have implications for our understanding of the origins of temperament in females, given the significant association found between DNA methylation of *NR3C1* and fearfulness in females. These findings support a malleable pathway to fearfulness that may be independent of heritable influences. In addition, this study offers a basic organizing perspective (i.e., temperament) through which research on epigenetics and early emotionality can be integrated. This study is, to our knowledge, the first to demonstrate an association between epigenetic processes and fearfulness in human infants. Temperament provides a developmental perspective on the “psychobiological study of individual differences in basic behavioral response styles or dispositional traits” (p. 395, Nigg, 2006). Temperamental traits are moderately stable, biologically-based patterns of engagement with one’s environment (Rothbart, 2012). Fearfulness, a trait associated with later risk for anxiety (Chronis-Tuscano et al., 2009; Buss, 2011), has traditionally been considered a behavioral outcome of heritable differences in stress responsivity (Kagan, 2013). However, the present findings suggest that individual differences in early fearfulness may arise, in part, through epigenetic modifications to the neuroendocrine system. These alterations to an individual’s stress response system lay the foundation for engaging with the environment (Bromer et al., 2013; Conradt et al., 2013, 2015), and suggest an alternative, experience-mediated pathway to the development of a fearful temperament.

Limitations of the current study must be considered when interpreting the results. First, there were no effects in the sample as a whole, and sex-specific findings were exploratory. In addition, these findings are correlational. There is substantial evidence that epigenetic processes are a mechanism of the effect of environmental influences on alterations in gene expression. It may be that methylation of *NR3C1* due to stress exposure early in development affects emotionality in infancy. Alternatively, early emotionality may affect methylation through the potential responses it evokes from caregivers (e.g., maltreatment; Romens et al., 2015). The sampling of buccal cells at 5-months also leaves open the possibility that environmental factors, including the mother-infant bond, occurring in the first months of life may affect results from the present study. Maternal sensitivity—a proxy for the mother-infant bond—was examined in relation to the variables of interest. No significant associations were found. The effect of prenatal maternal depression was also examined as a correlate of the variables of interest (data not shown) due to the frequent co-occurrence of depressive symptoms and stressful life events (Ertel et al., 2011). We found no significant associations for either male or female infants (results available upon request). Nevertheless, some aspects of early caregiving that were not measured in the present study may be affecting results. Furthermore, the modest number of women who reported stressful events during pregnancy, the majority of whom reported a single event, restricted our ability to specify whether the type (e.g., interpersonal) and/or timing (e.g., third trimester) of stressful events were associated with methylation of *NR3C1* or fearfulness. Finally, the absolute levels of methylation observed in this study were low, but remain consistent with previous research in early childhood (Oberlander et al., 2008; Parade et al., 2016).

The present study advances our understanding of the relation between prenatal stress, fearfulness, and adaptive changes of the glucocorticoid receptor gene. These findings expand on previous work by suggesting the possibility of an alternative, experience-dependent pathway to fearfulness in early life among females. Furthermore, by positioning findings within a temperament framework, the present study offers a basic organizing perspective through which future research on epigenetics and early emotionality can be interpreted and integrated. Understanding the perinatal mechanisms underlying the transmission of stress and manifestation of temperament will further clarify the developmental origins of risk for internalizing psychopathology.

## Author Contributions

All authors participated in the concept, design, implementation and analysis of the data, and take responsibility for the research reported. All authors participated in the drafting and revising of the manuscript and reviewed and approved the final submitted manuscript.

## Funding

This study was supported by the National Institute of Mental Health R01MH094609 (to CJM) and a Career Development Award from the National Institute on Drug Abuse 7K08DA038959-02 (to EC). The content is solely the responsibility of the authors and does not necessarily represent the official views of the National Institute of Mental Health, National Institute on Drug Abuse, or the National Institutes of Health.

## Conflict of Interest Statement

The authors declare that the research was conducted in the absence of any commercial or financial relationships that could be construed as a potential conflict of interest.
