# Supplementary material for: Prenatal Stress, Fearfulness, and the Epigenome: Exploratory Analysis of Sex Differences in DNA Methylation of the Glucocorticoid Receptor Gene
Source: Front Behav Neurosci. 2016 Jul 12;10:147. doi: 10.3389/fnbeh.2016.00147 (PMC4940423; doi:10.3389/fnbeh.2016.00147)
Supplement: Supplementary file 1 [file DataSheet_1.docx]

# Appendix
